# Electronic cigarette vapour increases virulence and inflammatory potential of respiratory pathogens

**DOI:** 10.1186/s12931-019-1206-8

**Published:** 2019-12-18

**Authors:** Deirdre F. Gilpin, Katie-Ann McGown, Kevin Gallagher, Jose Bengoechea, Amy Dumigan, Gisli Einarsson, J. Stuart Elborn, Michael M. Tunney

**Affiliations:** 1 Halo Research Group, School of Pharmacy, Queen’s University Belfast, 97 Lisburn Road, Belfast, Northern Ireland BT9 7BL UK; 2 Centre for Experimental Medicine, Queen’s University Belfast, 97 Lisburn Road, Belfast, Northern Ireland BT9 7BL UK

**Keywords:** Lung, Pathogen, E-cigarette, Cigarette, Cytokine, Virulence, Inflammation, Persistence

## Abstract

**Introduction:**

Bacteria have been extensively implicated in the development of smoking related diseases, such as COPD, by either direct infection or bacteria-mediated inflammation. In response to the health risks associated with tobacco exposure, the use of electronic cigarettes (e-cigs) has increased. This study compared the effect of e-cig vapour (ECV) and cigarette smoke (CSE) on the virulence and inflammatory potential of key lung pathogens (*Haemophilus influenzae, Streptococcus pneumoniae, Staphylococcus aureus* and *Pseudomonas aeruginosa*).

**Methods:**

Biofilm formation, virulence in the *Galleria mellonella* infection model, antibiotic susceptibility and IL-8/TNF-α production in A549 cells, were compared between bacteria exposed to ECV, CSE and non-exposed bacteria.

**Results:**

Statistically significant increases in biofilm and cytokine secretion were observed following bacterial exposure to either ECV or CSE, compared to non-exposed bacteria; the effect of exposure to ECV on bacterial phenotype and virulence was comparable, and in some cases greater, than that observed following CSE exposure. Treatment of A549 cells with cell signaling pathway inhibitors prior to infection, did not suggest that alternative signaling pathways were being activated following exposure of bacteria to either ECV or CSE.

**Conclusions:**

These findings therefore suggest that ECV and CSE can induce changes in phenotype and virulence of key lung pathogens, which may increase bacterial persistence and inflammatory potential.

## Background

Smoking is a risk factor for the development and progression of chronic lung diseases, such as chronic obstructive pulmonary disease (COPD) and asthma [1, 2]. Exposure to cigarette smoke initiates a cascade of tissue inflammatory responses and protease imbalances, which contribute to lung inflammation and aid establishment of chronic lung infection [3–5]. Electronic cigarettes (e-cigs) are widely perceived by the public as a safer alternative to tobacco smoking and their use has increased dramatically in recent years [6, 7]. Significant controversy exists around their use, dividing opinion amongst public health specialists [8, 9]. Since e-cigs contain fewer toxic chemicals, and in lower concentrations, than conventional cigarettes, they are viewed by some as a “lesser evil”. However, insufficient evidence regarding either their value as a smoking cessation tool or their safety compared to conventional cigarettes is currently available [10–12]. Of concern, recent reports have identified clusters of acute pulmonary disease associated with use of nicotine containing electronic cigarettes [13].

Bacteria, particularly *Haemophilus influenzae*, *Streptococcus pneumoniae*, *Staphylococcus aureus* and *Pseudomonas aeruginosa* have all been implicated in the development of smoking-related chronic lung disease, through both direct infection and bacteria-mediated inflammation [14]. Sequencing based studies have shown that these bacteria are associated with the development of a lung community skewed towards loss of diversity, and associated with declining lung function [15, 16] Although many studies have focused on the interaction between bacteria and host lung tissues, it is unclear how this complex interplay is affected by bacterial exposure to either conventional cigarette smoke or e-cigarette vapour. We hypothesize that such exposure may act as an environmental pressure on the respiratory pathogens, driving establishment of chronic lung infection through changes in bacterial phenotype and virulence, subsequent development of inflammation, and ultimately result in poorer clinical outcomes. Therefore, in this study we compared the effect of cigarette smoke extract (CSE) and e-cig vapour extract (ECVE) on the phenotype and virulence of respiratory pathogens.

## Methods

### Bacterial isolates

Isolates used in this study were obtained from the American Type Culture Collection (ATCC): *H. influenzae* (ATCC 49766), *S. aureus,* (ATCC 29213), *S. pneumoniae* (ATCC 49619) and *P. aeruginosa* (ATCC 27853). All isolates were stored at -80 °C prior to inoculation onto chocolate blood agar (*H. influenzae:* Oxoid, Basingstoke, UK) or blood agar (*S. aureus, S. pneumoniae, P. aeruginosa:* Oxoid, Basingstoke, UK) and incubated at 37 °C in 5% CO_2_ (*H. influenzae, S. pneumoniae),* or in air (*S. aureus, P. aeruginosa)*.

### Preparation of cigarette smoke and electronic cigarette vapour

#### Preparation of cigarette smoke extract (CSE)

CSE was prepared from Marlboro Red™ cigarettes (0.8 mg nicotine, 10 mg Tar; 10 mg carbon monoxide /cigarette), as previously described with minor modifications [17]. Cigarette smoke (35 ml) was drawn, using a sterile syringe, through 100 ml of appropriate culture medium every 15 s for 5 min. This action was repeated with either four, three, two or one cigarette per 100 ml of culture medium (termed 100, 75, 50 and 25% CSE, respectively). Following sterilisation by filtration through both 0.45 μm and 0.2 μm filters, the optical density_550nm_ was determined for all CSE solutions to ensure between batch consistency. All CSE exposed media was inoculated onto Mueller Hinton agar and incubated at 37 °C overnight to ensure sterility of the media prior to bacterial inoculation.

#### Preparation of E-cigarette vapor extract (ECVE)

ECVE was generated in identical fashion to CSE, except with a commercially available e-cigarette [Vapourlites™ (VL-EGO 650, (http://www.vapourlites.com/)] and using unflavoured e-liquid containing 10 mg/ml nicotine*.* Given the wide variety of e-cig devices currently available on the market, we chose one that at the time of study was a best –seller and widely available. Four, three, twice or once × 5 min vaping/100 ml of culture medium (termed 100, 75, 50 and 25%, ECVE respectively) was used. The resulting ECVE was then sterilised by filtration, and sterility of ECVE exposed media checked, as described above.

### Determination of total viable count (TVC) of bacteria following growth in CSE or ECVE

A suspension of 1 x 10^7^cfu of each bacteria (*H. influenzae, S. pneumoniae, S. aureus* and *P. aeruginosa*) was inoculated into 10mls culture media +/− 100, 75, 50 or 25% CSE/ECVE. Total viable counts were determined in triplicate at t = 0, 2, 4, 6, 24 and 48 h post inoculation as described previously and expressed as cfu/ml [18]. Bacterial growth in media, which had not been exposed to CSE/ECVE, was tested in parallel. Transmission electron micrograph (TEM) images were kindly prepared by Dr. Kathryn Whyte, EM Research Services, Newcastle University. Briefly, samples were fixed in 2% glutaraldehyde in Sorenson’s phosphate buffer, post-fixed in osmium tetroxide and dehydrated in graded acetone. They were then embedded in epoxy resin (TAAB premix medium) and polymerised for 24 h at 60 °C. Ultrathin sections (70 nm) were picked up on copper grids, stained with uranyl acetate and lead citrate before being imaged on a Hitachi HT7800 TEM with EMSIS camera.

### Growth of bacterial biofilm in CSE and ECVE

Biofilm formation of each isolate grown in media alone, or media exposed to either either 100% CSE or ECVE was determined by crystal violet staining of adherent cells after 24 h, as described previously [19].

### Effect of exposure to CSE/ECVE on bacterial virulence in the *Galleria mellonella* infection model

Changes in virulence of isolates in response to growth in media alone, or to media exposed to CSE/ECVE was determined using the *G. mellonella* infection model as described previously [20]. Following overnight growth in media +/− CSE/ECVE, the inoculum was washed by centrifugation and adjusted to 1 × 10^8^ cfu/ml in broth, to obtain a sub-lethal inoculum concentration, which both avoided immediate larval kill and allowed a change in % survival to be observed (Additional file 1: Table S1). Inoculation of larvae was carried out as previously described [21]. Briefly, for each test condition, batches of 10 larvae were inoculated with bacteria grown in the presence or absence of CSE or ECVE, or PBS, into the left, last set of pro-legs on each larvae prior to incubation at 37 °C in air for 24 h. Experiments were carried out in triplicate and % survival recorded.

### Development of resistance to antibiotics commonly used in the treatment of chronic lung infection

All isolates were inoculated in media alone, or media exposed to 100 or 50% CSE or ECVE. Following overnight incubation, each culture was adjusted to approximately 5 x10^6^cfu and inoculated into 10mls of fresh culture medium +/− CSE or ECVE. This serial passage was repeated daily for 12 days, with the MIC determined at 0, 3, 6, 9 and 12 days post inoculation by E-test® (BioMerieux, BioMerieux UK Ltd., Basingstoke, UK) in accordance with manufacturers instructions. Antibiotics tested were amoxicillin, co-amoxiclav, tetracycline, doxycycline, erythromycin, azithromycin and ciprofloxacin. At day 12, isolates in which resistance development had been observed were cultured in CSE/ECVE-free media for a further 12 days and MICs determined once more.

### Immune response to bacteria following exposure to CSE/ECVE

Human airway epithelial A549 cells (ATCC CCL-158) were passaged in complete medium [RPMI 1640, 10 μl/ml (v/v) penicillin/streptomycin solution, 10 μl/ml (v/v) HEPES buffer, 10% v/v foetal calf serum (Life Technologies, UK)] and incubated in 5% v/v CO_2_ at 37 °C. Bacterial infection of A549 cells was carried out by seeding cells into 24-well plates at a density of 2.5 x 10^5^cells/ml and overnight incubation until 70–90% confluency was achieved. Bacteria which had been grown for 24 h in media alone or media + 100% CSE or ECVE were added to serum-starved cells at a multiplicity of infection of 100 cfu/cell. Negative controls of PBS only were also included in each experiment. The viability of A549 cells under each treatment condition was determined at 2, 4 and 6 h post infection, by staining with Alamar Blue® (ThermoFisher UK Ltd., Paisley, UK) in accordance with the manufacturers instructions. Viability was determined by measurement of fluorescence at 600_nm_ and percentage viability calculated by fluorescence_sample_/fluorescence_control_ × 100.

At 0, 4 and 6 h post infection an aliquot of cell supernatant was removed and stored for cytokine analysis. All experiments were carried out in triplicate. Levels of IL-8, TNF-α and IL-1β were determined by ELISA (Peprotech, UK) in accordance with the manufacturers instructions, and standard curves generated using GraphPad Prism (version 5.00 for Windows, GraphPad Software, San Diego California USA). The above cell infection experiments were repeated, but with the addition of cell signaling inhibitors (BAY117085, SB203580, U0126 and SP600125, Tocris U.K.) which were added 1 h prior to bacterial infection of the cells, and levels of IL-8 and TNF-α in supernatants determined by ELISA (Additional file 1: Table S2).

### Statistical analyses

Differences in the growth of bacterial biofilm in CSE and ECVE were analysed using the Wilcoxon signed-rank test with Bonferroni’s adjustment for multiple comparisons [GraphPad Prism (version 6, GraphPad Software, San Diego California USA]. A one-way ANOVA test with Tukeys test for multiple comparisons was used to compare changes in *G.mellonella* following bacterial infection +/− CSE/ECVE exposure [R Environment version 3.3.1 (http://www.r-project.org)]. Changes in IL-8 and TNF-α +/− CSE/ECVE were analyzed by the Mann Whitney test, and the effect of pathway inhibitors, by pairwise comparison using Kruskal-Wallace test and Dunn’s test [R Environment version 3.3.1 (http://www.r-project.org)].

## Results

### Determination of TVC of bacteria following growth in CSE or ECVE

CSE or ECVE had no observable effect on the growth of any isolate tested, at any concentration, compared to growth of the isolate in media without CSE/ECVE. (Additional file 1: Figure S1). With higher concentrations of CSE, a slight lag in initial growth rate was observed, particularly with *H. influenzae*, but this was not evident at 24 h. Comparison of TEM images following exposure to either CSE or ECVE showed no gross physiological changes compared to bacteria grown in media alone, with the exception of *P.aeruginosa.* Exposure of *P.aeruginosa* to either CSE or ECVE resulted in increased numbers of cells in which the cytoplasm appeared to be partly detached from the cell wall (Additional file 1: Figure S2). However, this was not associated with any change in *P. aeruginosa* viability.

### Effect of CSE/ECVE on bacterial growth in biofilm

**Fig. 1:**
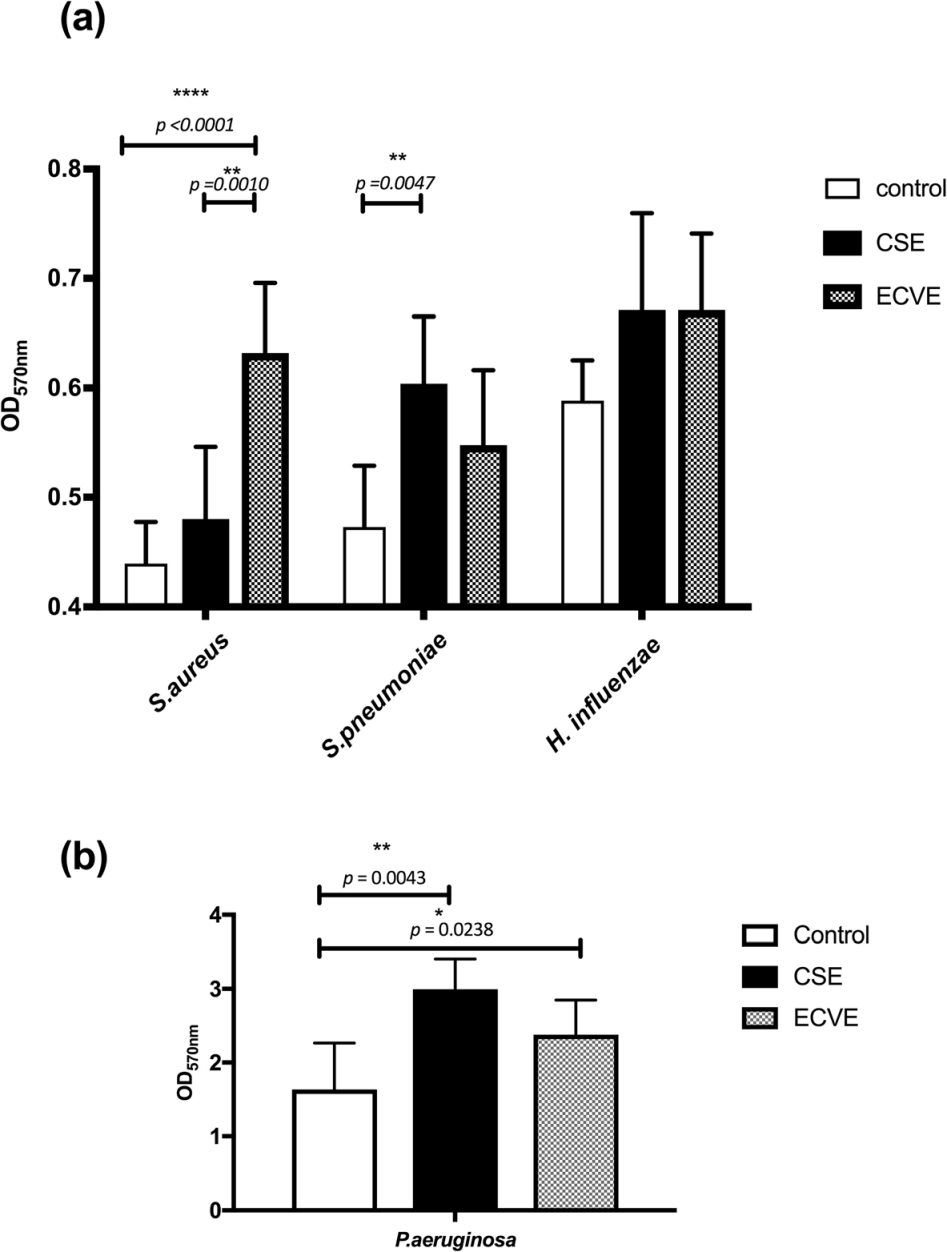
Effect of CSE and ECVE on biofilm formation. A trend towards increased biofilm formation was observed in all isolates, with statistically significant differences observed with (**a**) *S. aureus* + CSE/ECVE, *S. pneumoniae* + CSE and (**b**) *P. aeruginosa* + CSE/ECVE. The mean OD was calculated based on values from 4 replicates, repeated twice

Growth of isolates in culture medium containing CSE resulted in an increase in biofilm formation for all species compared to isolates grown in media alone, with statistically significant increases apparent for *S. pneumoniae* (*p* = 0.0047) and *P. aeruginosa* (*p* = 0.0043) (Fig. 1). A significant increase in biofilm formation was also observed for *S. aureus* cultured in media + ECVE (*p <* 0.001) compared to that in media alone. No difference was observed in biofilm formation in isolates cultured in CSE vs. ECVE, with the exception of *S. aureus* (*p* = 0.001) in which biofilm formation was higher in ECVE compared to CSE.

### Effect of bacterial exposure to CSE/ECVE on survival of *G. mellonella*

**Fig. 2:**
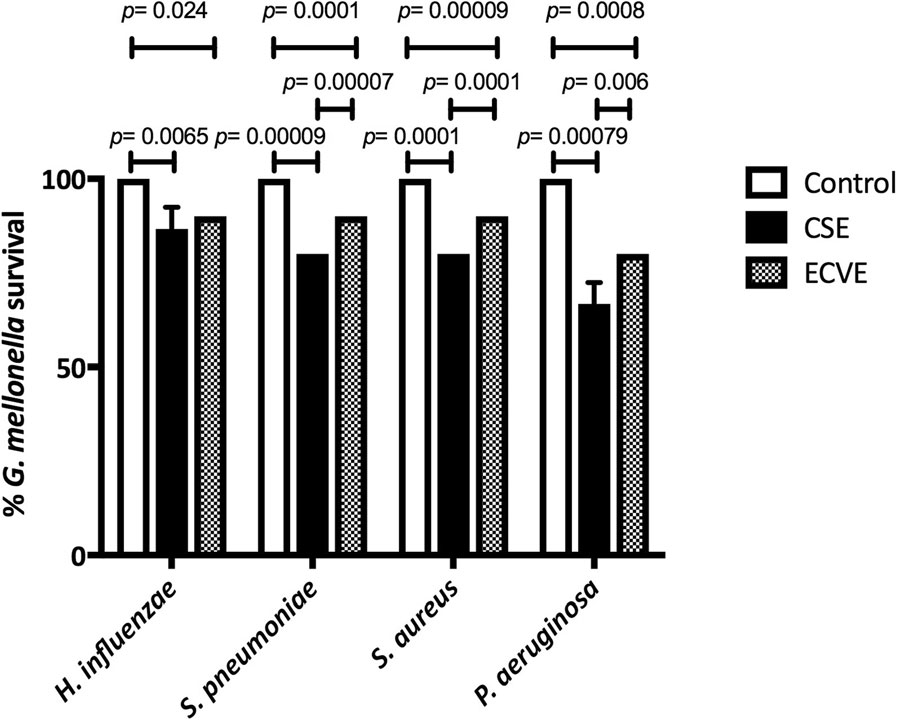
Effect of CSE and ECVE exposure on bacterial virulence in the *G. mellonella* infection model (*n* = 10). Larval survival decreased significantly in all isolates following exposure of isolates to both CSE and ECVE, compared to controls

We observed a statistically significant decrease in survival of *G. mellonella* infected with bacteria exposed to CSE or ECVE compared to larvae infected with bacteria not exposed to either CSE/ECVE (Fig. 2). The observed decrease was greater following bacterial exposure to CSE, compared to ECSE.

### Development of resistance to antibiotics commonly used in the treatment of chronic lung infection

The MIC of *P. aeruginosa* exposed to CSE to both tetracycline and doxycycline increased from 24 mg/ml and 48 mg/ml respectively, to > 256 mg/ml, within three days of exposure to CSE. This increase in MIC returned to original levels when isolates were cultured in the absence of CSE for 24 h, and the observed stability remained for the remaining 12 days of the experiment. No change in MIC of any other antibiotic was observed with the remaining isolates passaged in CSE or ECVE (Additional file 1: Table S3).

### Immune response to bacteria +/− CSE/ECV

**Fig. 3:**
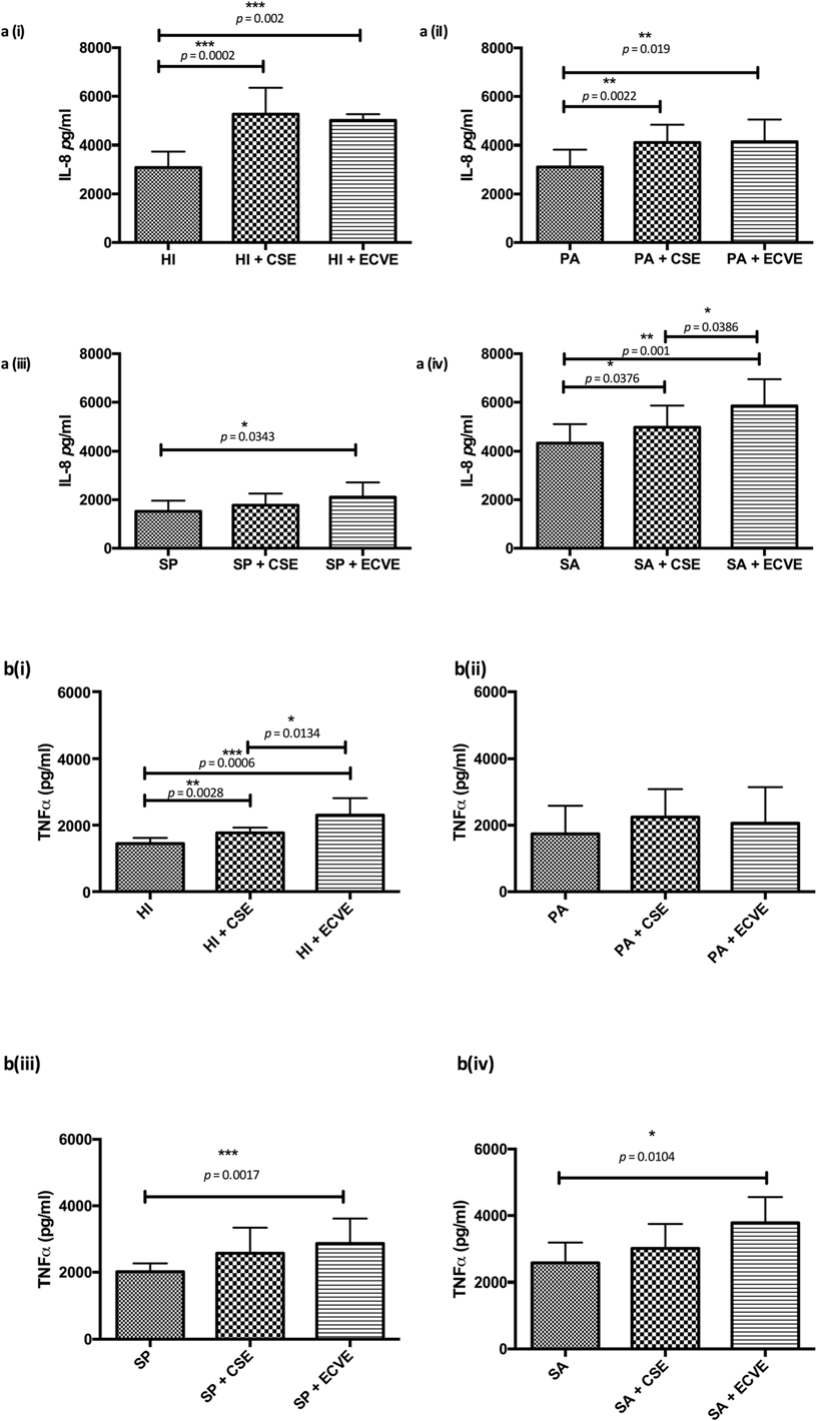
The effect of cigarette smoke extract (CSE) and electronic cigarette vapour (ECVE) exposure on the capacity of key lung pathogens (i) *H. influenzae* ATCC 49766 (HI), (ii) *P.aeruginosa* ATCC 27853 (PA), (iii) *S. pneumoniae* ATCC 49619 (SP) and (iv) *S.aureus* ATCC 29213 (SA), to stimulate (a) IL-8 (*n* = 9) and (b)TNF-α (n = 9) production from A549 cells

Exposure of A549 cells to bacteria exposed vs. bacteria not exposed to CSE resulted in a statistically significant increase in IL-8 secretion, with the exception of *S. pneumoniae* [*H. influenzae* (*p* = 0.0002); *P. aeruginosa* (*p =* 0.0022); *S. aureus* (*p =* 0.0372)] [Fig. 3(a)]. Exposure of bacteria to ECVE prior to A549 infection resulted in a statistically significant increase in IL-8 secretion with all bacteria + ECVE vs. bacteria not exposed to ECVE [*H. influenzae* (*p* = 0.0002); *P. aeruginosa* (*p =* 0.0019); *S. aureus* (*p =* 0.0372); *S. pneumoniae* (*p =* 0.0343)]. Levels of TNF-α were significantly increased in *H. influenzae* in response to CSE exposure (*p =* 0.0028) and in all bacteria exposed to ECVE with the exception of *P. aeruginosa* [*H. influenzae* (*p* = 0.0006); *S. pneumoniae* (*p =* 0.0017); *S. aureus* (*p =* 0.0104)] [Fig. 3(b)]. Viability of A549 cells remained at approximately 100% under each treatment condition and over the duration of the experiment, as determined by Alamar Blue® staining (Additional file 1: Figure S3).

**Fig. 4:**
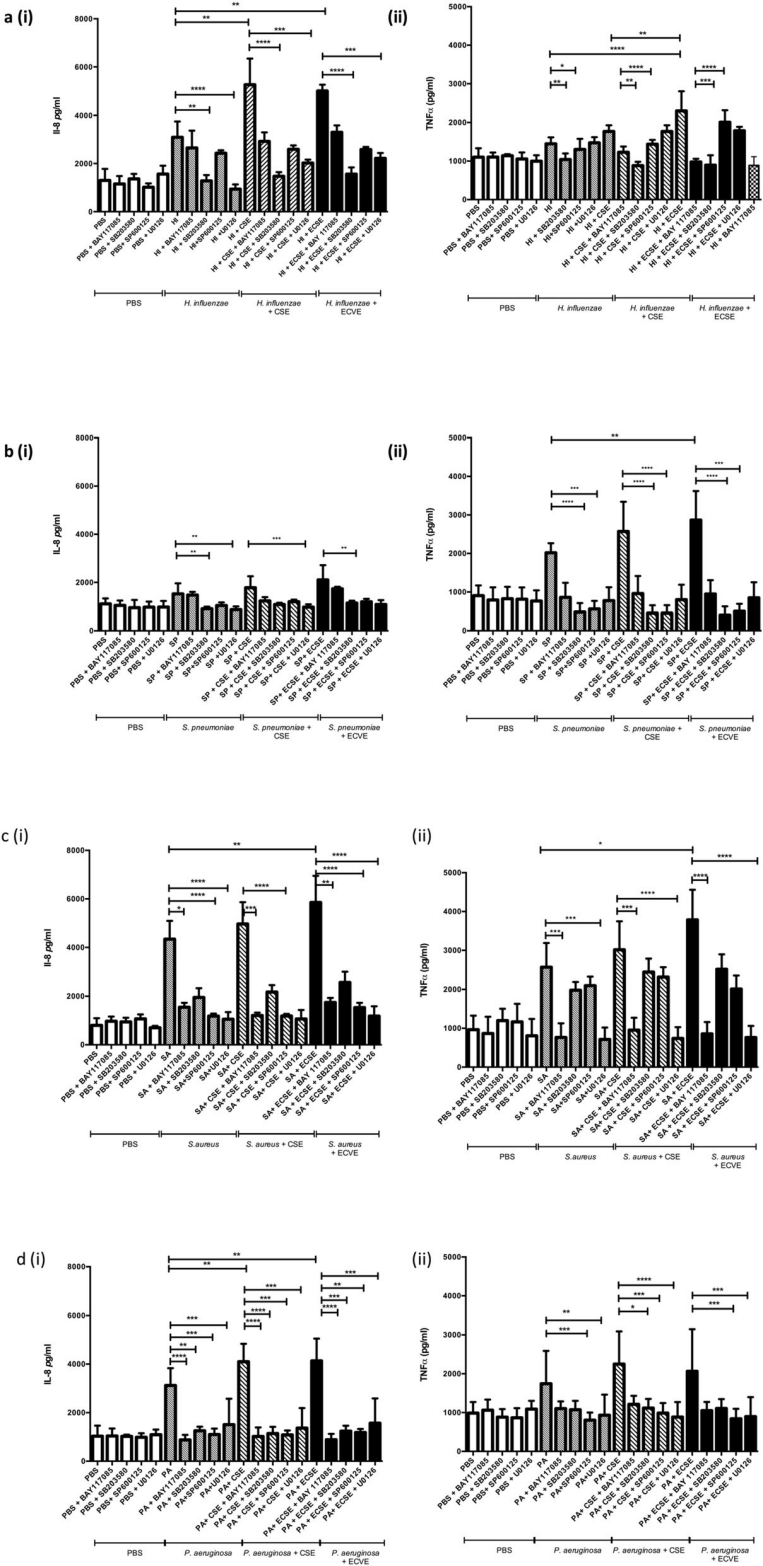
The effect of cigarette smoke extract (CSE) and electronic cigarette vapour (ECVE) exposure on the capacity of key lung pathogens to stimulate IL-8 [(**a**)-(**d**) (i)] and TNF-α [(**a**)-(**d**)(ii)]production from A549 cells (*n* = 8). Cell pathway signaling inhibitors were added to determine the contribution of each pathway to the cytokine production observed and the subsequent reduction in secretion of IL-8 or TNF- α measured. *P*-values are shown in (Additional file 1: Table S3)

The activation of NF-kB and MAP kinases, p38, ERK and JNK, is associated with the expression of inflammatory cytokines. To determine which one of these signaling pathways governed the increase in inflammation observed with ECVE-treated bacteria, infections were carried out in the presence of well-characterized pharmacological inhibitors. Use of pathway inhibitors resulted in a decrease in both IL-8 and TNF-α secretion by A549 cells following bacterial infection either alone, or following bacterial exposure to ECVE or CSE (Fig. 4, Table 1 and Additional file 1: Table S4). In general, the overall findings from these pathway inhibitor experiments show that the inflammatory pathway employed following bacterial exposure to ECVE was similar to that activated following infection with bacteria alone, or bacteria exposed to CSE.

**Table 1:** Production of IL-8 and TNF- α following treatment of A549 cells with pathway inhibitors, and infection with bacteria, or bacteria exposed to cigarette smoke extract or electronic cigarette vapour. Where a statistically significant reduction in cytokine was observed, this was denoted by “↓”

	Significant decrease (↓)in IL-8 following treatment of A549 cells with pathway inhibitors, and infection with bacteria +/− CSE or ECVE				Significant decrease (↓)in TNF-α following treatment of A549 cells with pathway inhibitors, and infection with bacteria +/− CSE or ECVE			
	NFκB inhibitor *(BAY117085)*	MAPK inhibitor *(SB203580)*	JNK inhibitor *(SP600125)*	MEK1/2 inhibitor *(U0126)*	NFκB inhibitor *(BAY117085)*	MAPK inhibitor *(SB203580)*	JNK inhibitor *(SP600125)*	MEK1/2 inhibitor *(U0126)*
*H. influenzae*		↓		↓	↓	↓		
*H. influenzae* + CSE		↓		↓	↓	↓		
*H. influenzae* + ECVE		↓		↓	↓	↓		
*S. pneumoniae*		↓		↓		↓	↓	
*S. pneumoniae* + CSE				↓		↓	↓	
*S. pneumoniae* + ECVE		↓				↓	↓	
*S.aureus*	↓		↓	↓	↓			↓
*S.aureus* + CSE	↓		↓		↓			↓
*S.aureus* + ECVE	↓		↓	↓	↓			↓
*P.aeruginosa*	↓	↓	↓	↓			↓	↓
*P.aeruginosa* + CSE	↓	↓	↓	↓		↓	↓	↓
*P.aeruginosa* + ECVE	↓	↓	↓	↓			↓	↓

## Discussion

In this study, changes in bacterial phenotype associated with virulence were observed following exposure to ECVE. In some cases the observed phenotypic changes were less than those observed with CSE-exposed bacteria (e.g. with virulence in the *G. mellonella* model). However, in general, there was little difference in the effect on exposure of bacteria to CSE or ECVE, with exposure to either resulting in increased virulence and inflammatory potential of the bacterial isolates.

Several studies have suggested an effect of ECVE on cultured lung cells, ranging from increased inflammation, measured by increased cytokine production, to changes in the microvasculature [22–24]. Increased cytokine production and evidence of lung injury has also been observed following exposure of mice to e-cig vapour and nicotine, together with a reduced capacity to clear either bacterial (*S. pneumoniae*) or viral (H_1_N_1_ Influenza) infection [23, 25, 26]. These findings suggest an inflammatory lung environment similar to that observed following cigarette smoking. Many e-cig users have previously been cigarette smokers; therefore, it is difficult to attribute any changes in lung function to e-cigs alone. However, perhaps driven by concerns over cigarette safety, many adolescents who have never smoked, are now taking up vaping [27], resulting in evidence of an association between e-cigarette use or exposure, and increased asthma exacerbations [28, 29]. There is therefore a need to understand the long-term impact of e-cigarette use and second hand ECV exposure, particularly on the lung health of vulnerable populations [12].

Bacterial colonization and infection of the airways is a contributing factor to lung function decline across a range of chronic lung diseases and a recognized risk of tobacco smoke exposure [30]. However, the extent to which cigarette smoke, or ECVE drives the establishment of bacterial colonization and aids persistence of these bacteria has not been extensively studied in all key pathogens implicated in chronic lung disease. *H. influenzae, S. pneumoniae, P. aeruginosa* and *S. aureus* are consistently associated with lung function decline, increased severity of disease and increased rate of exacerbation in chronic lung diseases in which smoking also plays an important role [31, 32]. Establishment of biofilm by these pathogens is a significant virulence determinant in the pathophysiology of chronic lung disease, and is associated with establishment and persistence of infection, resistance to antibiotics and evasion of the host immune system. In this study, biofilm formation increased in all isolates in response to both CSE and ECVE. Furthermore, the degree of biofilm formation observed following exposure of bacterial isolates to either CSE or ECVE, was similar and suggests that bacterial exposure to either CSE or ECVE may promote bacterial adhesion, biofilm formation and thus establishment of persistent infection. This reflects previous studies, which demonstrated similar findings following CSE exposure of lung (*S. aureus*, *P.aeruginosa* and *S. pneumoniae*) [33–39] and oral pathogens (*Streptococcus gordonii*, *Porphyromonas gingivalis* and *Candida albicans*) [40–42]. In all cases, genes associated with biofilm formation were found to be up-regulated, and this was linked to oxidative stress resultant from CSE exposure. Changes were also observed in expression of genes encoding for bacterial cell surface structures, resulting in increased bacterial adhesion to epithelial cells. MRSA exposed to CSE had increased hydrophobicity and altered surface charge, which resulted in increased adherence to epithelial cells and decreased bacterial susceptibility to antimicrobial peptides, respectively [35]. In the case of *P. gingivalis*, increased expression of fimbrial proteins induced TLR2 hyposensitivity and hence altered immune responses [41]. The effect of ECVE was not investigated in these studies, and further work will be required to determine if the observed increases in biofilm following ECVE exposure, occur by similar mechanisms. In this study, there was limited evidence of structural change by electron microscopy, following exposure of bacteria to either CSE or ECVE. Future work will therefore more fully investigate changes in bacterial transciptomes following exposure to vape or tobacco smoke.

Increased biofilm formation subsequent to CSE/ECVE bacterial exposure is suggestive of increased isolate virulence, and this hypothesis was further explored in the *G. mellonella* model. Numerous studies have shown that microbial pathogenesis and bacterial virulence are comparable in humans, mice and *G. mellonella* [21]. For the purposes of this study, it provided a high-throughput and cost-effective means by which changes in bacterial virulence could be assessed [43–45]. Statistically significant decreases in larvae survival (assumed to be consistent with increased bacterial virulence), were observed for all bacteria exposed to CSE, and for all bacteria exposed to ECVE, except *H. influenzae*. Mammalian models of lung infection will be required to more fully assess changes in host pathology following infection with CSE/ECVE exposed bacteria; however, our aim in this study was to assess gross changes in bacterial virulence.

A particularly striking finding of this study was the change in lung inflammation observed following infection of A549 cells with bacteria exposed to either CSE or ECVE. Dysregulation of the lung inflammatory response is a hallmark of chronic lung disease, such as COPD, where it is persistent, observed long after exposure to cigarette smoke has ceased, and attributed to bacterial colonization [46]. With the exception of *S. pneumoniae,* IL-8 secretion from A549 cells was significantly increased in all isolates following infection with bacteria exposed to CSE and ECVE, compared to infection with non-CSE/ECVE exposed bacteria. Of particular note, was that there was no difference observed between levels of IL-8 produced following infection with bacteria + CSE vs. bacteria + ECVE, with the exception of *S.aureus*. In this case, exposure to ECVE resulted in increased IL-8 levels compared to CSE. Levels of TNF-α were similarly increased following ECVE exposure with *H. influenzae, S. pneumoniae* and *S. aureus.* These data indicate that bacteria exposed to CSE promote a greater inflammatory response in A549 cells than in non-exposed bacteria, but that this is closely matched and in some cases exceeded by the level of inflammation observed following exposure to ECVE. Altered immune responses, which promote bacterial persistence, have previously been observed with *S. pneumoniae,* following airway cell-CSE exposure [47, 48] and with CSE- exposed MRSA [33]. MRSA exposure to ECVE has also been described as altering immunomodulatory cytokines in the airways of mice [49]. Our findings expand on this work to show that exposure of other key respiratory pathogens to both CSE and, in particular, ECVE, has the potential to modulate host response to infection and we speculate that this could contribute to the increased inflammation and bacterial persistence characteristic of smoking-related chronic lung disease. The epithelial cell-line A549 were considered to be suitable for this study since the epithelium is the major source of lung immunomodulatory factors and is hence critical in the modulation of inflammatory diseases such as COPD and bronchiectasis [50]. Furthermore, they are well characterized and standardized, allowing for rigorous comparison of bacterial infections. Future studies will more fully analyse the host response to CSE/ECVE exposed bacteria in a range of primary cell cultures, but this is outside the scope of the present study.

Addition of a range of immune pathway inhibitors suggested that the cell-signalling pathway utilized in response to infection is dependant on the bacterial species involved. Furthermore, the results did not indicate that increased cytokine production in response to bacterial exposure to ECVE was occurring via an alternative cell-signaling pathway, compared to bacterial infection alone or CSE-exposed bacteria. Moreover, bacterial CSE/ECVE exposure enhanced the immunomodulatory effect observed. Increased activation of both NFκB and MAPK signaling pathways have been implicated in the pathogenesis of COPD and asthma, with NFκB upregulation further associated with steroid insensitivity [51], but the potential contribution of bacterial infection to this pathway is still poorly understood. Our findings clearly indicate that these pathways may be further up-regulated by exposure of key lung pathogens to CSE or ECVE. The bacterial lung community is complex and increased airway inflammation subsequent to bacterial exposure to CSE/ECVE is likely to be mediated via a range of signaling pathways. Understanding each of these, and their respective contribution to inflammation in vivo may provide insight into potential therapies to reduce the effects of persistent bacterial-induced inflammation.

A recurring theme of this study is the similarity observed in the effect of exposure to CSE compared to ECVE on bacterial phenotype and virulence. CSE was generated in accordance with previously published and accepted protocols: however, this is a potential limitation of this study. In order to ensure comparability, CSE and ECVE were prepared using a similar method. This may not represent a true reflection of differences between smoking and vaping: e.g. it fails to take account of the differences in puffing topography (puff duration and flow rate) between conventional and electronic cigarettes, and between individuals [52]. E-cigarette users take larger and longer puffs, compared to conventional cigarette users, which may increase nicotine delivery. Our model may therefore underestimate the exposure of respiratory pathogens to ECVE [53]. Our current protocol is also based on a one-off exposure to CSE/ECVE, and used a brand of e-cigarettes with no added flavour: however, flavourings and e-cigarettes additives (such as PG/VG) have been associated with changes in the bronchial epithelia and impairment in respiratory innate immunity [54, 55]. Further studies are therefore required to investigate the effect of both common e-cigarette flavourings and long-term exposure of bacteria to CSE/ECVE. Furthermore, only reference isolates were used in this study and further work investigating a wider range of clinical isolates is required.

## Conclusions

Exposure of respiratory pathogens to e-cigarette vapour induced changes in phenotype and virulence, which may increase bacterial persistence and inflammatory potential. These changes were similar, and in some cases exceeded, those observed following bacterial exposure to cigarette smoke and suggest that there is little difference between the effect of CSE and ECVE. There is therefore an urgent need for further robust clinical studies investigating and clarifying the long-term effect of e-cigarette use on both airway cells and respiratory pathogens to enable a better informed judgment to be made regarding their safety.

## Supplementary information

**Additional file 1.** Supplementary figures and tables.

## Data Availability

All data generated or analysed during this study are included in this published article and its supplementary information files.
