# Supplementary material for: Electronic cigarette vapour increases virulence and inflammatory potential of respiratory pathogens
Source: Respir Res. 2019 Dec 18;20:267. doi: 10.1186/s12931-019-1206-8 (PMC6918581; doi:10.1186/s12931-019-1206-8)
Supplement: Supplementary file 1 — Additional file 1. Supplementary figures and tables. [file 12931_2019_1206_MOESM1_ESM.docx]

Supplementary data:

Supplemental data Figure 3. Viability of A549 cells following infection with (i) *Staphylococcus aureus* (SA) (ii) *Streptococcus pneumoniae* (SP) (iii) *Haemophilus influenzae* (HI) and (iv) *Pseudomonas aeruginosa* (PA). Bacteria were cultured in either Mueller Hinton broth (MHB), or MHB + Cigarette smoke extract (CSE) or MHB + Electronic cigarette vapour extract (ECVE). Viability was determined by Alamar Blue® staining, and expressed as a percentage viability compared to control (A549 cells only). All experiments were carried out in quadruplicate.

**Table 1. Bacteria and concentration of inoculum used to inject *G. mellonella* larvae.**

| Bacteria | Concentration of inoculum (cfu/ml) |
| --- | --- |
| *P. aeruginosa* | 1 x 10^1^ |
| *S. pneumoniae* | 1 x 10^6^ |
| *H. influenzae* | 1 x 10^6^ |
| *S. aureus* | 1 x 10^8^ |

| Inhibitor | Chemical name | Biological activity | Concentration (µg/ml) |
| --- | --- | --- | --- |
| BAY117085 | (2E)-3-[[4-(1,1-Dimethylethyl)phenyl]sulfonyl]-2-propenenitrile | Irreversible inhibitor of TNF-α-stimulated IκBα phosphorylation; leads to decreased NF-Κb. | 2.5 |
| SB203580 | 4-[5-(4-Fluorophenyl)-2-[4-(methylsulphonyl)phenyl]-1H-imidazol-4-yl]pyridine hydrochloride | Selective inhibitor of p38 mitogen-activated protein kinase (MAPK). | 2 |
| U0126 | 1,4-Diamino-2,3-dicyano-1,4-bis[2-aminophenylthio]butadiene | Potent and selective non-competitive inhibitor of mitogen-activated protein kinase kinase (MAPKK). Inhibits MEK-1 and MEK-2. | 5 |
| SP600125 | Anthra[1-9-cd]pyrazol-6(2H)-one | Selective JNK inhibitor. Competitively and reversibly inhibits JNK1, 2 and 3. | 5 |

**Table 2. Cell signaling pathway inhibitors used in this study, their biological activity and the concentration used.**

**Table 3: MIC values of antibiotics commonly used in the treatment of respiratory infection (ug/ml) before and after 12 days exposure to cigarette smoke.**

*S – sensitive, R –resistant; - denotes that susceptibility testing is not recommended as the species is a poor target for therapy with that particular agent.*

| **Antibiotic** | **Day of passage** | **MIC (µg/ml)** | | | |
| --- | --- | --- | --- | --- | --- |
|  |  | ***P. aeruginosa***  **(ATCC 27853)** | ***S. aureus***  **(ATCC 29213)** | ***S. pneumoniae***  **(ATCC 49619)** | ***H. influenzae***  **(ATCC 25238)** |
| **Tetracycline** | Day 1 | 24 (-)  >256 (-) | 0.125 (S)  0.125 (S) | 0.75 (S)  0.75 (S) | 1 (S)  1 (S) |
|  | Day 12 |  |  |  |  |
| **Doxycycline** | Day 1 | 48 (-)  >256 (-) | 0.064 (S)  0.064 (S) | 0.125 (S)  0.25 (S) | 1 (S)  1 (S) |
|  | Day 12 |  |  |  |  |
| **Co-amoxiclav** | Day 1 | >256 (-)  >256 (-) | 0.38 (-)  0.38 (-) | 0.064 (-)  0.064 (-) | 0.125 (S)  0.125 (S) |
|  | Day 12 |  |  |  |  |
| **Amoxicillin** | Day 1 | >256 (-)  >256 (-) | 0.75 (-)  0.75(-) | 0.064 (-)  0.047 (-) | 0.25 (S)  0.25 (S) |
|  | Day 12 |  |  |  |  |
| **Erythromycin** | Day 1 | 96 (-)  96 (-) | 0.19 (S)  0.19 (S) | 0.125 (S)  0.094 (S) | 4 (S)  4 (S) |
|  | Day 12 |  |  |  |  |
| **Azithromycin** | Day 1 | 24 (-)  24 (-) | 0.12 (S)  0.12 (S) | 1 (S)  0.75 (S) | 4 (R)  4 (R) |
|  | Day 12 |  |  |  |  |
| **Ciprofloxacin** | Day 1 | 0.25 (S)  0.25 (S) | 0.50 (S)  0.38 (S) | 1 (S)  0.75 (S) | 0.032 (S)  0.032 (S) |
|  | Day 12 |  |  |  |  |

**Table 4. IL-8 and TNFα production by A549 cells, following infection with bacteria exposed to cigarette smoke extract (CSE) or electronic cigarette vapour extract (ECVE), and addition of various cell signaling pathway inhibitors. P values obtained below outline show the significance of reduction and were calculated by pairwise comparison using Kruskal-Wallace test and Dunn’s test.**

|  | **Reduction in IL-8 (*p* value)** | | | | **Reduction in TNF-α (*p* value)** | | | |
| --- | --- | --- | --- | --- | --- | --- | --- | --- |
|  | *BAY117085* | *SB203580* | *SP600125* | *U0126* | *BAY117085* | *SB203580* | *SP600125* | *U0126* |
| ***H. influenzae*** | 1 | **4.90E-02** | 1 | **5.20E-05** | **2.60E-03** | **3.90E-02** | 1 | 1 |
| ***H. influenzae* +CSE** | 0.87 | **4.30E-07** | 0.17 | **4.00E-04** | **1.50E-02** | **3.00E-05** | 0.57 | 1 |
| ***H. influenzae* +ECVE** | 1 | **1.90E-06** | 0.39 | **6.30E-04** | **9.10E-04** | **1.40E-04** | 1 | 0.26 |
| ***S. pneumoniae*** | 1 | **9.10E-03** | 0.64 | **3.40E-03** | 0.15 | **4.70E-05** | **7.60E-04** | 6.07 |
| ***S. pneumoniae* +CSE** | 1 | 0.09 | 1 | **6.60E-03** | 0.23 | **1.50E-04** | **1.20E-04** | 0.07 |
| ***S. pneumoniae* +ECVE** | 1 | **8.90E-03** | 1 | 0.26 | 0.31 | **2.80E-05** | **4.00E-04** | 0.06 |
| ***S.aureus*** | 0.03 | 0.45 | **1.30E-05** | **1.80E-06** | **3.70E-04** | 1 | 1 | **3.00E-04** |
| ***S.aureus* + CSE** | **1.20E-03** | 0.84 | **1.80E-04** | 0.07 | **1.20E-03** | 1 | 1 | **7.00E-05** |
| ***S.aureus* + ECVE** | **8.00E-03** | 0.7 | **5.60E-05** | **1.10E-06** | **5.20E-05** | 1 | 0.11 | **1.50E-05** |
| ***P.aeruginosa*** | **3.10E-08** | **5.40E-03** | **1.20E-03** | **1.10E-03** | 1 | 1 | **1.40E-03** | **9.00E-03** |
| ***P.aeruginosa*+CSE** | **3.90E-06** | **5.20E-05** | **6.70E-04** | **3.30E-04** | 0.21 | **0.03** | **6.70E-04** | **1.30E-04** |
| ***P.aeruginosa*+ECVE** | **1.40E-08** | **5.90E-04** | **4.90E-03** | **8.10E-04** | 0.19 | 0.48 | **1.80E-03** | **1.80E-03** |

*P values ≤0.05 are considered significant and are denoted in green, with p values greater than 0.05 denoted in red.*
